# Association between in-country migration and HIV infection among transgender women from northeastern Brazil: a respondent-driven sampling survey

**DOI:** 10.1186/s12889-024-17956-6

**Published:** 2024-02-23

**Authors:** Beo Oliveira Leite, Laio Magno, Francisco Inacio Bastos, Ines Dourado

**Affiliations:** 1 Collective Health Institute, Federal University of Bahia, Av. Basílio da Gama, s/n, Campus Universitário do Canela, 40110-040 Salvador, Bahia Brazil; 2 Life Sciences Department, Bahia State University, Campus1, Salvador, Bahia Brazil; 3 Oswaldo Cruz Foundation, Rio de Janeiro, RJ Brazil

**Keywords:** Transgender women, “Travestis”, In-country migration, HIV, Brazil, RDS

## Abstract

**Background:**

Migration is common among transgender women (TGW), often driven by the desire to escape stigma, find acceptance, establish new connections, access body modifications, or enter new avenues of sex work. Given the heightened mobility of TGW, they are mostly vulnerable to human immunodeficiency virus (HIV) due to migration. This study aimed to evaluate the association between in-country migration and HIV infection among TGW in Northeast Brazil.

**Methods:**

The DIVAS was a cross-sectional, multicity study investigating risk behaviors and sexually transmitted infections (STI) among TGW in 2016–2017. A total of 864 TGW were recruited through respondent-driven sampling from three capital cities in Northeast Brazil. Logistic regression estimating odds ratios (OR) and 95% confidence intervals (CI) was used to assess the relationship between in-country migration and HIV infection.

**Results:**

The prevalence of HIV among TGW was 24.5%, 21.4% among those aged 18–34 and 36.1% among those ≥ 35 years old. In-country migration increased the odds of HIV infection among TGW aged 18–34 years (OR = 1.84; 95%CI:1.04–3.27) and even higher among those aged ≥ 35y old (OR = 3.08; 95%CI:1.18–8.04).

**Conclusions:**

These data reinforce the pressing need for public health policies that provide comprehensive access and strategies for demand creation for HIV/AIDS prevention and care for TGW who are already highly vulnerable to infection.

## Background

In 2022, approximately 39 million people worldwide were estimated to be living with human immunodeficiency virus (HIV) infection, and 1.3 million people acquiring new infections [1]. Additionally, a 5% increase in new infections was reported in Latin America between 2010 and 2021, with 2.2 million people living with HIV (PLWH) in 2021. Moreover, 93% of PLWH aged 15–49 years in Latin America were from key populations [2]. This term is adopted by the Joint United Nations Programme on HIV/AIDS (UNAIDS) to refer to populations at high risk and vulnerability to infection: drug users, sex workers, sexual partners of sex workers or other members of key populations, gays, and other men who have sex with men (MSM), and transgender women (TGW) [2].

The worldwide prevalence of HIV among TGW is estimated to be 19.9% [3]. In Latin America, the prevalence in the general population aged ≥ 15 years is estimated at ∼ 0.4% while among TGW is ∼ 25.9% (20.0–31.8) [3]. In Brazil, this prevalence varies from 9.0 to 40.9% depending on the study sampling process and region [3, 4]. The worldwide odds of infection are 85.3-times higher compared to the general population of reproductive age (15–49 years old) [5]. High vulnerability to HIV can be explained by behavioral (e.g., unprotected sex), programmatic (e.g., barriers to access health services, technologies, and strategies), and sociostructural factors (e.g., stigma and discrimination) [6–9].

Because they confront the rules and norms prevalent in mainstream society, TGW are often marginalized and occasionally expelled from home by their own families and forced into migratory flow between cities in their own country or outside the country. This arrangement of trajectories involves the confrontation of stigma, discrimination, and violence in various spheres of socialization such as family, school, work, public and private spaces, and health services [10–14]. These obstacles are often responsible for greater migratory flow among TGW seeking better living conditions, job opportunities, and spaces of greater acceptability [11].

Migration is a social phenomenon of permanent or temporary human displacement that results from factors responsible for attracting individuals to a new location (e.g., employment, quality of life, and social support) and factors responsible for repelling individuals from their place of origin (e.g., discrimination, inequality, conflicts, and diseases) [15]. Internal or in-country migration refers to displacement among cities in the same country, whereas international or external migration refers to displacement among countries [16]. In Brazil, a country with continental dimensions and different cultures, in-country migration can involve displacements over long distances comparable to international migration and is therefore subject to similar obstacles.

In-country and international migration is observed among TGW [10–12, 17]. Regardless of the type, such displacements can significantly affect the lives and health of TGW. Immigrants within a country or internationally may present with greater poverty, unemployment, social exclusion, less access to health services, and negative health outcomes (including HIV/AIDS) in comparison to the resident population [18–21]. International immigrants may be subjected to even greater obstacles (e.g., language differences [which is not the case in Brazil, an unusual exception worldwide for a continent sized country where a single language is spoken by every citizen], cultural differences, absence/scarcity of support networks, exclusion, or transphobia) [16, 22].

Although some studies have investigated international migration among TGW [12, 17, 23–29], few have analyzed the effects of in-country migration. To date, no study has evaluated the effects of in-country migration on HIV infection in Brazil. In-country migrants, particularly TGW, have a higher risk of HIV infection than native populations increasing their existing vulnerability. We aimed to analyze the association between in-country migration and HIV infection among TGW in Northeast Brazil.

## Methods

The DIVAS research was a cross-sectional, multicity survey conducted between October 2016 and July 2017. It was formally called the “Nationwide study of behavior, attitudes, practice and prevalence for HIV, syphilis and hepatitis B and C among *travestis* and other transgender women in 12 Brazilian municipalities”. This cross-sectional study is a part of the larger DIVAS research study which was conducted across three capital cities in the northeast region of Brazil i.e., Salvador, Recife, and Fortaleza.

### Study population

The study inclusion criteria were self-identifying as a t*ravesti*, trans woman or other female gender identity, having been registered as male at birth, and having had at least one sexual intercourse in the last 12 months. In Latin American countries, the terms “t*ravestis*” or “trans women” are commonly used by individuals and communities themselves too. *“Travesti”* is a Latin American ethno-cultural term which is especially used in Brazil and has no translation in the English language. Participants ≤ 18 years old and those under the influence of alcohol or other drugs at the time they would be putatively interviewed were excluded from the study.

### Data collection and sampling

TGW were recruited from three capitals in Northeast Brazil in which the study was conducted: Salvador, Recife, and Fortaleza. Respondent-driven sampling (RDS) was used as the sampling method. It is a chain-link sampling method generally used for hard-to-reach populations, based on recruiting individuals by their peers within their network of contacts. Focus groups and in-depth interviews were conducted with TGW, and initial participants (seeds) were selected. Each seed and subsequent participant received three coupons to invite another TGW from their social contact networks (referral chains). This methodology presupposes financial incentives: the primary incentive refers to reimbursement for participation and the secondary incentive refers to reimbursement by the invitation of each recruited pair [30].

The study was conducted through social movements and common places of coexistence of TGW (e.g., bars, nightclubs, public squares, and private spaces) to identify seeds. Seven seeds were chosen from Salvador, six from Fortaleza, and five from Recife. Each seed received three coupons to invite three other TGW for sample collection, and the chain of invites continued until the required sample size was achieved. All TGW, including the recruited seeds, were given a referral to the research centers. They signed an informed consent on participation, responded to the survey questionnaire, underwent rapid testing, and received counseling both before and after the test. In cases where HIV infection and/or other sexually transmitted infections (STI) were identified, the TGW received support until they were successfully linked to health services to ensure the continuity of treatment.

Data were collected through face-to-face interviews using a standardized pretested questionnaire conducted by interviewers in a space reserved exclusively for this purpose. The questionnaire was organized into 11 blocks: sociodemographic information; knowledge about STI, viral hepatitis, and access to condoms; healthcare; HIV, syphilis, and hepatitis testing; discrimination, violence, and human rights violations; sexual behavior; alcohol and other drugs; body modifications; mental health; involvement with the criminal justice system; and social support, resilience, and trans-pride. Rapid HIV antibody testing was performed according to the Brazilian Ministry of Health Standards of Care [31]. The methodological details have been detailed in Bastos et al. (2018) [32].

### Study variables

**Fig. 1:**
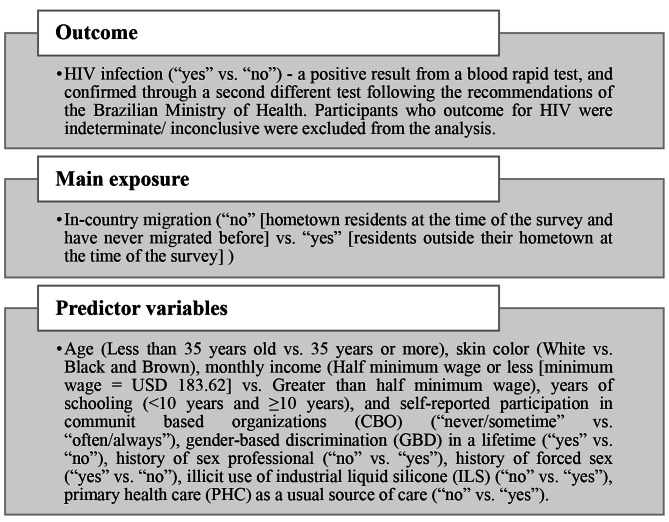
Study variables

The study variables are presented in Fig. 1.

### Data analysis

Descriptive statistics for in-country migration are summarized as contingency tables and plots. The association between HIV infection, in-country migration, and covariates were assessed using Pearson’s χ^2^ test, and adjusted odds ratios (AOR) were estimated using binomial logistic regression. Age was considered as a potential effect modifier of the main association and regression models were estimated for each age group of 18–34 and ≥ 35y old. Covariates with a *p*-value < 0.20 were included in the multivariate analyses for models addressing both people aged 18-34y and ≥ 35y old as outcomes. The final models were selected using the backward strategy, considering parsimony and the best fit for each iteration round. The variables were removed from the model considering a significance level at a *p*-value < 0.05, a percentage change from the main effect of the OR to the confounding adjustment of the main association, or theoretical importance based on the available literature. The Hosmer-Lemeshow goodness-of-fit test verified the final adequacy of the model.

All data analyses were weighted as suggested by the RDS-II estimator [33], which performs an adjustment according to the degree of homophily and sample size of the social contact network. In our study, missing data were not deemed significant, and were disregarded and excluded from the tables. The weights were calculated using RDS Analyst 0.42, and other analyses were performed for the survey data using Stata software, version 15.0 (Stata Corporation, College Station, USA).

## Results

A total of 864 TGW were included in the study, but 34 TGW chose not to perform the rapid HIV test in our study and two did not answer questions on in-country migration, hence, 828 TGW were included for the final analysis. Most TGW (55.6%) were aged ≥ 25 years, self-declared to have a black or brown skin color (84.3%), and had a monthly income higher than half the minimum wage (i.e., about 65.5% [minimum wage = USD 183.62]). They had ≥ 10 years of schooling (54.5%), most did not participate in activities of CBO (77.1%), had suffered gender-based discrimination at least once in their lifetime (87.3%), worked as sex professional at least once in their lifetime (68.7%), suffered sexual violence (61.2%), and did not use industrial liquid silicone as implants (76.6%). Attendance at a primary healthcare consultation was reported by 33.0% (described by them as their usual source of care), and 50.4% reported a syphilis infection in the past (Table 1).

**Table 1:** Distribution of study variables according to migration within the country among TGW from Northeast Brazil, 2017

Variables	Total		In-country migration ^b^			
			No		Yes	
	*n* ^c^	% (95%CI)^c^	*n* ^c^	% (95%CI) ^c^	*n* ^c^	% (95%CI) ^c^
**HIV infection** ^**a**^						
No	632	75.5 (71.1–79.5)	421	81.2 (76.2–85.3)	210	66.7 (58.5–74.1)
Yes	198	24.5 (20.5–28.9)	103	18.7 (14.7–23.8)	94	33.3 (25.9–41.5)
**Age**						
18–34 years	675	78.5 (74.2–82.1)	444	79.4 (74.0–84.0)	229	76.5 (69.5–82.3)
35 years or more	189	21.5 (17.9–25.7)	101	20.6 (16.0–26.0)	88	23.5 (17.7–30.5)
**Ethnicity/skin color**						
White	136	15.7 (12.6–19.4)	83	15.5 (11.9–20.0)	53	16.4 (11.1–23.5)
Black and Brown	681	84.3 (80.6–87.4)	435	84.5 (80.0-88.1)	244	83.6 (76.5–88.9)
**Monthly income**						
Half minimum wage or less	293	34.5 (30.1–39.1)	209	38.0 (32.5–43.8)	83	28.8 (22.0-36.6)
Greater than half minimum wage	571	65.5 (60.9–69.9)	336	62.0 (56.2–67.5)	234	71.2 (63.4–78.0)
**Schooling**						
10 years or more	473	54.5 (49.7–59.2)	292	55.9 (49.9–61.7)	180	51.5 (43.4–59.5)
Up to 9 years	378	45.5 (40.8–50.3)	245	44.1 (38.3–50.1)	132	48.5 (40.5–56.6)
**Participation in CBOs**						
No	628	77.1 (73.2–80.6)	407	78.3 (73.4–82.5)	219	74.8 (67.8–80.7)
Yes	233	22.9 (19.4–26.8)	137	21.7 (17.5–26.6)	96	25.2 (19.3–32.2)
**Gender-based discrimination**						
No	92	12.7 (9.5–16.8)	64	15.8 (11.4–21.4)	27	7.5 (4.0-13.6)
Yes	772	87.3 (83.2–90.5)	481	84.2 (78.6–88.6)	290	92.5 (86.4–96.0)
**History of sex professional**						
No	203	31.3 (26.9–36.1)	156	36.5 (30.9–42.5)	46	21.4 (14.8–30.0)
Yes	661	68.7 (63.9–73.1)	389	63.5 (57.5–69.1)	271	78.6 (70.0-85.2)
**History of forced sex**						
No	331	38.8 (34.3–43.5)	224	41.3 (35.7–47.2)	106	34.8 (27.4–43.1)
Yes	533	61.2 (56.5–65.7)	321	58.7 (52.8–64.3)	211	65.2 (56.9–72.6)
**Use of industrial liquid silicone**						
No	633	76.6 (72.4–80.3)	458	85.6 (80.9–89.4)	173	60.3 (52.4–67.7)
Yes	228	23.4 (19.7–27.6)	86	14.3 (10.6–19.1)	142	39.7 (32.3–47.6)
**PHC as a usual source of care**						
No	559	67.0 (64.5–71.2)	347	66.1 (60.4–71.3)	211	69.3 (61.8–76.0)
Yes	304	33.0 (28.8–37.5)	197	33.9 (28.7–39.6)	106	30.7 (24.0-38.2)

^a^ 34 TGW did not perform HIV rapid tests; ^b^ No response on in-country migration to 2 TGW; ^c^ Weighted by RDSII

Of the total number of participants, 24.5% tested positive for HIV: 21.4% were aged 18–34 years, and 36.1% were aged ≥ 35 years old. Moreover, 545 TGW (63.5%) reported living in the municipality of origin, and 317 (36.5%) had migrated within the country (Table 2).

**Table 2:** Description of HIV infection and in-country migration according to age among TGW from Northeast Brazil, 2017

Variables	Total		18–34 years		35 years or more	
	*n* ^c^	% (95%CI) ^c^	*n* ^c^	% (95%CI) ^c^	*n* ^c^	% (95%CI) ^c^
**HIV infection** ^a^						
No	632	75.5 (71.1–79.5)	521	78.6 (73.7–82.9)	111	63.9 (53.4–73.3)
Yes	198	24.5 (20.5–28.9)	135	21.4 (17.1–26.3)	63	36.1 (26.7–46.6)
**In-country migration** ^**b**^						
No	545	63.5 (58.7–67.9)	444	64.3 (58.9–69.4)	101	60.3 (50.2–69.7)
Yes	317	36.5 (32.1–41.3)	229	35.7 (30.6–41.1)	88	39.7 (30.3–49.8)
**Time of residence in the current city**						
Less than 5 months	193	50.0 (42.8–57.3)	158	55.8 (47.1–64.1)	35	32.6 (21.5–46.1)
5 years or more	191	50.0 (42.7–57.2)	117	44.2 (35.9–52.9)	74	67.4 (53.9–78.5)
**Main reason to leave the city of origin**						
Looking for work/ Improving quality of life	159	53.7 (45.3–62.0)	114	52.8 (43.0-62.4)	45	57.1 (40.7–72.0)
Being close to friends/family/partner	79	27.9 (21.2–35.8)	62	29.0 (21.2–38.4)	17	24.1 (13.1–40.1)
Rejection/ Discrimination/ Violence	24	10.2 (6.1–16.6)	15	8.7 (4.5–16.4)	9	15.3 (6.8–31.0)
Gender readjustment	20	5.4 (3.0-9.6)	15	6.2 (3.2–11.4)	5	2.8 (0.7–10.6)
To study	6	2.7 (0.6–11.3)	4	3.3 (0.7–14.6)	2	0.8 (0.2–3.4)

^a^ 34 TGW did not perform HIV rapid tests; ^b^ No response on in-country migration to 2 TGW; ^c^ Weighted by RDSII

The residence time in the current city was < 5 years for 50.0% of the participants. The residence time was significantly longer among TGW aged ≥ 35 years (*p* = 0.004). For TGW aged 18–34 years and ≥ 35 years, the main reason for leaving the city of origin was to look for work or better quality of life (52.8% and 57.1%, respectively), followed by being closer to friends, family, or partner (29.0% and 24.1%), rejection, discrimination and violence (8.7% and 15.3%), body modifications (6.2% and 2.8%), and to seek educational opportunities (3.3% and 0.8%; Table 2).

The bivariate analysis revealed that being a migrant increased the odds of HIV infection in both age groups: 18–34 years (OR = 1.94; 95% CI:1.11–3.38) and ≥ 35y old (OR = 2.77; 95% CI:1.17–6.57) compared with TGW residents of the city of origin. Other variables significantly associated with HIV infection in the 18–34 years age group were years of schooling (*p* = 0.018) and participation in CBO activities (*p* = 0.046). Variables associated with HIV infection in the ≥ 35 years age group were low for black and brown skin color (*p* = 0.037) and high for past and current sex work (*p* = 0.009; Table 3).

**Table 3:** Bivariate analysis of factors associated with HIV infection among TGW in Northeast Brazil, 2017

Variables	18–34 years			35 years or more		
	%^a^	*p*-value	OR (95%CI) ^a^	%^a^	*p*-value	OR (95%CI) ^a^
**In-country migration**		0.019			0.019	
No	16.8			26.6		
Yes	28.1		1.94 (1.11–3.38)	50.0		2.77 (1.17–6.57)
**Ethnicity/skin color**		0.841			0.024	
White	20.4			56.6		
Black and brown	21.9		1.09 (0.45–2.63)	30.0		0.33 (0.12–0.88)
**Monthly income**		0.178			0.126	
Half minimum wage or less	17.4			25.0		
Greater than half minimum wage	23.5		1.45 (0.84–2.51)	41.3		2.11 (0.80–5.54)
**Schooling**		0.018			0.191	
10 years or more	16.5			27.4		
Up to 9 years	27.6		1.93 (1.11–3.33)	41.3		1.86 (0.73–4.76)
**Participation in CBOs**		0.046			0.796	
No	23.3			34.7		
Yes	13.6		0.52(0.27-<1.00)	37.4		1.13 (0.46–2.78)
**Gender-based discrimination**		0.449			0.093	
No	17.1			17.2		
Yes	22.0		1.37 (0.60–3.11)	39.3		3.11 (0.78–12.34)
**History of sex professional**		0.209			0.009	
No	16.3			14.2		
Yes	23.7		1.60 (0.77–3.34)	45.7		5.11 (1.41–18.54)
**History of forced sex**		0.496			0.856	
No	19.3			35.0		
Yes	22.6		1.22 (0.69–2.16)	36.8		1.08 (0.46–2.58)
**Use of industrial liquid silicone**		0.065			0.077	
No	19.7			27.2		
Yes	30.9		1.83 (0.96–3.49)	45.5		2.24 (0.91–5.52)
**PHC as a usual source of care**		0.984			0.239	
No	21.4			40.1		
Yes	21.5		1.00 (0.56–1.80)	27.8		0.57 (0.23–1.45)

^a^ Weighted by RDSII

The model with the best fit indicated that being a migrant increases the odds of HIV infection by 84% (AOR = 1.84; 95% CI:1.04–3.27) for interviewees in the 18–34 years age group and 208% (AOR = 3.05; 95%CI:1.15–8.07) in the ≥ 35 years age group compared with TGW residents of the city of origin (Table 4).

**Table 4:** Multivariate adjustment of the association between in-country migration and HIV infection among TGW in Northeast Brazil, 2017

18–34 years				35 years or more			
Empty model	AdjOR ^a^	*p*-value	95%CI	Empty model	AdjOR ^a^	*p*-value	95%CI
**In-country migration**	1.94	0.020	1.14–3.37	**In-country migration**	2.77	0.021	1.17–6.57
**Full model**				**Full model**			
**In-country migration**	1.77	0.070	0.95–3.27	**In-country migration**	3.04	0.042	1.04–8.89
Adjusted by schooling. participation in CBOs, monthly income, use of industrial liquid silicone.				Adjusted by ethnicity/skin color, monthly income, schooling; gender-based discrimination, history of sex professional, use of industrial liquid silicone.			
**Final Model**				**Final Model**			
**In-country migration**	1.84	0.036	1.04–3.27	**In-country migration**	3.05	0.025	1.15–8.07
Adjusted by schooling, participation in CBOs, monthly income.				Adjusted by ethnicity/skin color; history of sex professional.			

^a^ Weighted by RDSII

## Discussion

The estimated prevalence of HIV infection among TGW was very high and especially high, as is evident from other studies on older TGW. There are two key factors to be observed here. First, similar to any infection putatively acquired by repeated unprotected interactions, the prevalence tends to increase over time. Second, dedicated programs targeting the health of TGW, and other sexual and gender minorities are recent in Brazil and in most countries [34]. In this sense, in addition to individual risks, birth cohort effects must be considered. Older cohorts did not benefit from referrals to treatment centers. In many settings, they could not find a single health center tailored to their needs and specificities.

A meta-analysis of 15 countries estimated the global HIV prevalence to be 19.1% among TGW. Another meta-analysis conducted in the United States estimated an HIV prevalence rate of 18.8% [35]. Other studies from countries such as China, Iran, and Cambodia estimated a prevalence of less than 15% [36–38]. One must observe here the challenge of the proper categorization of TGW and men. In several countries, this category may not exist as such (and has usually been confounded with other sexual minorities or ignored). Most likely, world estimates tend to underestimate actual infection rates. In Brazil, estimates of the transgender female population may vary but seem to be acceptable as rough estimates [39].

A study of TGW from eight African countries reported a prevalence of HIV closer to that in our study (25.4%) [40]. In some Latin American countries, such as Uruguay (21.5%) nd Argentina (34.1%),the HIV prevalence among TGWs is also similar [41, 42]. In Brazil, the estimated prevalence of TGW in a meta-analysis was 33.1% [5]. Other studies from different regions of the country, such as in Rio Grande do Sul (25%) and Rio de Janeiro (31.2%) have also identified a similar HIV prevalence to the TGW reported in the present study [43, 44]. Despite advancements in technology and science, the adoption of new HIV prevention strategies such as, pre-exposure prophylaxis in health services of the Brazilian National Health System [45], and the implementation of decentralization policies for HIV prevention [46, 47], these achievements do not extend to the Brazilian TGW. Numerous studies have highlighted the challenges TGW face in accessing the healthcare system in Brazil, with transphobia being a significant factor [13, 48–52]. It is crucial to emphasize that additional intersectional factors contribute to a lack of access to healthcare facilities, for example, the majority of TGW in this study were Black or Brown with less than 10 years of schooling, amplifying their vulnerability to HIV [9].

Regarding migration, younger TGW spent less time in new cities compared to the ones they had lived in before. The main reason that led them to travel in both groups was the need to seek work and attempt to improve their quality of life. Our findings confirm anecdotal reports that for TGW, the search for contexts offering a higher quality of life is a concern that often arises as an urgent requirement when they are young but that usually persists throughout life. Migration is part of the life trajectory of this population and is done with the intention to escape violence and discrimination, and search for acceptability and a higher quality of life [12, 23]. The lifelong quest for a decent life is not easily fulfilled in many contexts, therefore, comprehensive structural modifications are required.

In-country migration increased the odds of HIV infection in TGW aged 18–34 years old, and such an effect may be even higher among those aged ≥ 35 years old. Since policies for the prevention and treatment of HIV were effectively implemented in the early 2000s [53], older TGW had a greater risk of exposure to the epidemic (i.e., more than two decades earlier) when such policies were absent for all practical purposes.

Although younger TGW may have had opportunities to access strategies and services after the timely implementation of proper policies, as there is no cure for the infection yet, HIV prevalence remains very high. Available policies do not meet their needs, are not necessarily implemented in the best way possible, and may not translate into actual changes toward safer behaviors and practices. Implementation science has documented several limitations in various fields [53]. In-country migration appears to be a key factor in aggravating vulnerability.

Migration, both in-country and internationally, is responsible for increasing individual vulnerability because it tends to decrease access to health services and technologies [20, 54, 55], mainly in the context of HIV/AIDS prevention, management, and care services [21, 56–59]. Even when TGW do not face barriers related to differences in culture or language, they are usually unaware of the health service network and lack support networks when they arrive in a new city within the same country. It takes time and willpower to get used to the new context and to form new bonds and learn how to better navigate health and social welfare systems. Particularly for TGW, for whom obstacles to access health services and HIV prevention strategies are already present [13, 50, 60, 61], living outside the place of origin may be, at the same time, tempting, as happens to any land of actual or imaginary opportunities, and a putative source of vulnerability.

In a cross-sectional study involving TGW from Lima, Peru, internal and international migration significantly increased the infection rates of gonorrhea and chlamydia. However, the prevalence of HIV infection did not differ significantly between migrants and non-migrants [26]. Another cohort study involving TGW from New York City revealed an association between international migration and HIV infection [25]. Because the incubation times of acute (e.g., gonorrhea) and chronic (e.g., HIV) infections differ markedly, cross-comparisons comprising different timings are far from simple and linear.

Migratory displacements can break social bonds, i.e., the TGW who have migrated may lose support networks and foster resilience, which is a key factor in HIV/AIDS prevention. These skills and resources are essential for challenging stigma and fostering discussions about risk, prevention, and testing [62].

In a qualitative study by Barrington et al. (2018) that investigated sexual relations between men and Latino transmen and women in North Carolina, the United States, identified that dialogue about HIV, HIV testing, and sexual behaviors among international migrant networks was superficial or rare, which could make them more vulnerable to infection [63].

Furthermore, the context in which immigrant TGW comprise their new place of residence, both in their country of origin and abroad, may influence their risks, protective behaviors, attitudes, and practices. Immigrants tend to emulate the dynamics and customs of the recently established networks. This is a consequence of the conscious and unconscious efforts to adapt [20, 64, 65]. A TGW who newly comes into a network of contacts in which risk behaviors are prevalent may emulate them. The seminal work of Howard Becker documented such processes in detail among many different people, from jazz musicians to people who are drug-dependent, and sometimes members of the same social networks [66].

Nuttbrock and Hwahng (2017) reported that foreign transwomen residing in New York City had an increased chance of engaging in sex work and were not protected from receptive anal sex. In another cross-sectional study involving Hirjas from Bangladesh, participants who migrated outside their country of origin were less likely to report condom use during anal sex in the last intercourse, consistent condom use during anal sex with new clients in the last week, or consistent condom use during anal sex with regular clients in the last week [17].

In a qualitative study by Bianchi et al. (2014) that investigated sexual relations between men who had sex with transmen and transwomen from Bogotá, pointed out that migration within a country or internationally resulted in social isolation for many participants who lost their previous support networks and thereby engaged in sex work. Participants reported that they faced difficulties in negotiating safe practices with their clients, their knowledge about HIV and other STI was limited, and HIV testing was infrequent, making this population particularly vulnerable.

This study had some limitations. The cross-sectional design prevented us from defining a temporal relationship between the response variable and the main exposure. Unfortunately, this is an intrinsic limitation of any straightforward chain referral process. Studies combining chain referral and capture recapture seem to be exciting alternatives; however, their empirical effectiveness in exploring the directionality of associations remains to be fully demonstrated, in addition to their seminal contributions to the estimation of population size [67]. Although there is a lag between the data collection period and the publication of these results, this delay is not considered a significant limitation. Publications focusing on TGW populations remain relatively scarce in the literature compared to those on the general population. Throughout this period, there have been no notable advancements in addressing transphobia in Brazil, and HIV prevalence remains high in this demographic region. Moreover, strategies to mitigate the impact of migratory dynamics on the accessibility of HIV prevention measures for these populations are lacking. Therefore, it is imperative to investigate these barriers.

This analysis was not aimed at identifying whether HIV infection occurred before or after migration, but rather at assessing vulnerability to infection in groups with higher mobility. RDS and other nonprobability sampling methods pose challenges for statistical inference [68]. However, such limitations did not prevent the generation of important information about the network of interviewees, as implemented in several studies conducted worldwide [37, 69–73].

Moreover, the questionnaire in this study was not designed for an in-depth assessment of the questions under analysis, and such limitations preclude the analysis of possible confounding factors and mediators. This limitation is a key feature of large multicity studies, where the trade-off between comprehensiveness and in-depth assessment of some aspects is a permanent challenge, not only in terms of theoretical assumptions or psychometric properties, but also more frequently associated with mundane difficulties such as budget and time constraints.

## Conclusion

The present study revealed a high prevalence of HIV among TGW and a significantly higher probability of infection among migrants in both age groups compared with interviewees who remained in their city of origin. Such disproportionate HIV infection rates can be explained by trajectories of lower or higher vulnerability. In addition to being subjected to social exclusion secondary to gender discrimination, migration may reinforce inauspicious living conditions against migrants’ expectations, making the population even more vulnerable.

Governmental agencies at all levels, activists, NGOs, and civil society should establish sound advocacy coalitions aimed at strengthening and implementing public policies that provide TGW with effective access to services and technologies to deliver care and prevention for this population. This is a major health and ethical imperative.

## Data Availability

The datasets analyzed in this study are available from the Harvard Dataverse repository, 10.7910/DVN/UINIOB.
